# Identification of population substructure among Jews using STR markers and dependence on reference populations included

**DOI:** 10.1186/1471-2156-11-48

**Published:** 2010-06-14

**Authors:** Jennifer B Listman, Deborah Hasin, Henry R Kranzler, Robert T Malison, Apiwat Mutirangura, Atapol Sughondhabirom, Efrat Aharonovich, Baruch Spivak, Joel Gelernter

**Affiliations:** 1 Department of Psychiatry, Yale University School of Medicine, New Haven, CT, USA; 2 VA Connecticut Healthcare System, West Haven Campus, West Haven, CT, USA; 3 Department of Psychiatry, Columbia University College of Physicians and Surgeons, NY, USA; 4 Departments of Psychiatry and Genetics and Developmental Biology, University of Connecticut School of Medicine, Farmington, CT, USA; 5 Chulalongkorn Faculty of Medicine, Bangkok, Thailand; 6 Sackler Faculty of Medicine, Tel Aviv University, Tel Aviv, Israel; 7 New York State Psychiatric Institute, NY, USA; 8 Dept Epidemiology, Mailman School of Public Health, Columbia University, NY, USA; 9 Department of Genetics, Yale University School of Medicine, New Haven, CT, USA; 10 Department of Neurobiology, Yale University School of Medicine, New Haven, CT, USA

## Abstract

**Background:**

Detecting population substructure is a critical issue for association studies of health behaviors and other traits. Whether inherent in the population or an artifact of marker choice, determining aspects of a population's genetic history as potential sources of substructure can aid in design of future genetic studies. Jewish populations, among which association studies are often conducted, have a known history of migrations. As a necessary step in understanding population structure to conduct valid association studies of health behaviors among Israeli Jews, we investigated genetic signatures of this history and quantified substructure to facilitate future investigations of these phenotypes in this population.

**Results:**

Using 32 autosomal STR markers and the program STRUCTURE, we differentiated between Ashkenazi (AJ, N = 135) and non-Ashkenazi (NAJ, N = 226) Jewish populations in the form of Northern and Southern geographic genetic components (AJ north 73%, south 23%, NAJ north 33%, south 60%). The ability to detect substructure within these closely related populations using a small STR panel was contingent on including additional samples representing major continental populations in the analyses.

**Conclusions:**

Although clustering programs such as STRUCTURE are designed to assign proportions of ancestry to individuals without reference population information, when Jewish samples were analyzed in the absence of proxy parental populations, substructure within Jews was not detected. Generally, for samples with a given grandparental country of birth, STRUCTURE assignment values to Northern, Southern, African and Asian clusters agreed with mitochondrial DNA and Y-chromosomal data from previous studies as well as historical records of migration and intermarriage.

## Background

The genetics of Jewish populations, particularly that of Ashkenazi Jews, has been studied extensively to answer questions of human evolutionary, historical, and medical significance [1-11]. Human evolutionary or anthropological studies have typically focused on mitochondrial DNA (mtDNA) or Y-chromosomal data, because the absence of recombination in these regions of the genome allows researchers to infer past human behaviors and evolutionary events such as migrations, founder events, population bottlenecks or expansions, relative male and female contributions to an admixed population, marriage practices, and mode of transmission of languages [12-15]. However, medical research necessitates the use of autosomal data. The depth of data collection and the necessary characterization of subpopulations to control for population stratification during case-control association studies provide a unique resource to augment mtDNA and Y-chromosomal studies and to facilitate the investigation of selection events. For population groups in which group identification is based on cultural practices rather than geographic origin (such as religion for the Jews or Spanish language for Hispanics), the hazard in neglecting such structure may be particularly great in medical genetics studies [16-19].

Y-chromosomal and mtDNA studies of Jewish populations and their local host populations have, at times, provided conflicting results, but can be summarized as supporting the following: 1. Almost all Jewish populations are derived from Middle Eastern ancestral populations [3,8,11,20-23]; 2. Bottleneck events have had an effect on the gene pools of Jewish populations [2, 4-6, 21]; 3. Local female contribution was significant in the establishment of Yemenite, Ethiopian, and Indian Jewish populations [6]; 4. Local male contribution has been less significant for the establishment of most Jewish populations [23], but may have contributed more to Ashkenazi than to non-Ashkenazi populations [3,8,20,22].

Several large-scale studies using autosomal markers demonstrated substructure among European populations, specifically non-Jewish Northern European, non-Jewish Southern European, and Ashkenazi Jews [24-26]. Additionally, based on haplotype analysis, recent mtDNA surveys of Ashkenazi and non-Ashkenazi Jewish populations and non-Jewish host populations demonstrated substructure among Jewish populations [6,27]. Although Jewish populations other than Yemenite, Ethiopian, and Indian have not been entirely endogamous, local admixture from host populations, the amount of which varies among populations, has generally occurred at low levels. These historical events may contribute to population structure and stratification that should be taken into consideration in the analysis of data from association studies.

Using thousands of SNPs and principal components analysis (PCA), Seldin et al [25], Price et al [24], and Tian et al [26] found "Northern" and "Southern" components in non-Jewish European populations, which followed a gradient from Northwest Europe to Southeast Europe or North to South, depending on the SNPs used. However, they also reported that both Ashkenazi and Sephardic Jewish samples showed, on average, more than 85% ancestry from the "Southern" component, regardless of grandparental country of birth. They concluded that this reflects a Middle Eastern origin of both Southeast Europeans and Ashkenazi Jews, which both admixed subsequently to varying extents with populations already occupying Europe. A recent study analyzing a large set of autosomal SNPs [10] using PCA demonstrated that not only is it possible to cluster Ashkenazi Jews separately from non-Jewish Europeans but also that the number of Ashkenazi Jewish grandparents determined where a sample fell on the PCA plot relative to non-Jewish Europeans. Recently, using a large number of STRs and several clustering methods, Kopelman et al [28] showed that four Jewish populations (Tunisian, Moroccan, Turkish, and Ashkenazi) clustered together and intermediate to other European and Middle Eastern populations. In all cases, the authors attributed these clustering patterns to the partial and shared Middle Eastern ancestry of Jews.

Middle Eastern ancestry may be a common factor among Jewish populations; however, the majority of Jewish populations have been located outside of the Middle East for up to 2000 years. As is the case with other highly mobile human populations there has been historically documented gene flow between Jewish populations and local host populations. In addition, because these are populations defined, in part, by religion, gene flow into Jewish populations is a product of conversion as well as marriage. Thus, there should be genetic admixture in Jewish subpopulations that reflects, in part, their migratory histories and may contribute to current genetic differences among Jewish populations. It is known that detecting and quantifying recent admixture is dependent on the time since divergence of the putative parental populations as well as the number and information content of markers. Because clustering algorithms are also dependent on the *relative *differences between populations, the context of a sample in a given analysis (i.e., the extent of its difference from samples of other populations included in the analysis) can affect clustering patterns. This aspect of the process of population substructure detection may be overlooked in case control association studies and may affect results if not taken into consideration. Based on this, we hypothesized that the presence or absence of putative parental populations in a STRUCTURE analysis would affect the ability to detect substructure in Jewish populations and differences between Jewish populations.

To address this question thoroughly prior to conducting association studies of health behaviors among Israeli Jews, we examined population structure in Jewish populations of European, African, Middle Eastern, Central and South Asian origin. We genotyped 526 subjects, recruited in Israel, with 32 genome-wide unlinked microsatellite markers (STRs). To identify potential population structure in the Jewish population being studied, we also genotyped 254 individuals from self-identified Chinese, Thai, Ethiopian Jewish, African American, and European American samples using the same markers. The Jewish populations sampled here are not comprised of various percentages of discreet ancestral populations. Our premise is that Jewish populations originated in the Middle East but, subsequent to and in the course of long-range migrations, accumulated input from local host populations, each with its own migratory history. We include in our analysis genotypic data from present-day populations whose ancestry serves as a proxy for those populations that might have contributed once Jewish populations migrated out of the Middle East. Our results are of interest both to infer unknown and correlate with known aspects of Jewish history and for their theoretical implications for detecting substructure in seemingly homogenous populations. They are also of important applied interest for studying health-related phenotypes in our sample of Israeli Jews. To our knowledge, this is the first study to incorporate proxy parental groups into analysis of structure of a Jewish sample, as well as the first to investigate variation among and ancestry of world-wide Jewish populations with autosomal markers.

Each of the Israeli subjects provided self-reported country of birth, country of birth of parents and grandparents, world region of family origin (not necessarily the same as country of birth of grandparents), whether they considered themselves to be Ashkenazi (as defined by respondents), Sephardic (similarly self-defined), mixed, other or none, and whether they, their parents, and grandparents had been born Jewish (also self-defined). A common practice in the medical and non-medical literatures is to subsume Jews of Spanish, Balkan, Middle Eastern, African, and Asian descent under the term "Sephardic", but since this term implies Spanish origin, it is imprecise and unclear. Further, due to continuous changes in the acceptability and applicability of the term, "Sephardic" among Israelis [29,30], medical and genetic studies involving Israeli participants increasingly refer to subjects as either "Ashkenazi" (AJ) or "non-Ashkenazi" (NAJ) [31-34]. Below, we also follow that nomenclature. This expands on work we first presented in 2008 [35].

## Results

### Population Differentiation: group affiliation

**Figure 1:**
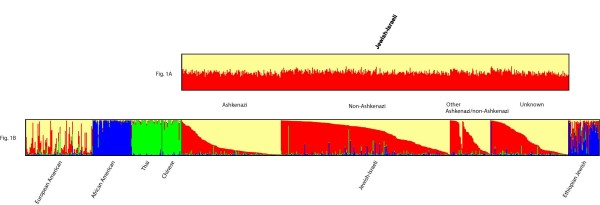
**STRUCTURE plots with and without world-wide samples**. a. STRUCTURE plot of European-American (EA) and Jewish-Israeli samples when K = 2. Each vertical line represents an individual with ancestry components shown as different colors. Self-identified group affiliation (Ashkenazi, Non-Ashkenazi, Other, Ashkenazi/non-Ashkenazi (for individuals with one parent from each group), or Unknown) is listed for Jewish-Israeli samples in between Fig. 1a and Fig. 1b. The order of Jewish-Israeli individuals is the same in figures 1a and 1b. b. STRUCTURE plot of European-American (EA), African American (AA), Thai, Chinese (Asian), Jewish-Israeli, and Ethiopian Jewish (EJ) samples when K = 4. Each vertical line represents an individual with ancestry components shown as different colors.

When there is no detectable substructure in a sample, after using the program STRUCTURE 2.2 [36,37] each individual will have nearly equal assignment values to each assumed population, giving the appearance that each individual is entirely and nearly equally admixed; when this pattern is observed, the result is not meaningful in terms of actual detection of structure [38]. When the mixed Jewish sample was analyzed alone, using STRUCTURE, the assignment values for K = 2 through K = 4 yielded no detectible substructure (Fig. 1a). When EA, AA and Asian samples were added to the analysis (with the effect of establishing parental populations for clustering), AJ was assigned to Southern 0.23%, Northern 0.73%, Asian 0.02%, and African 0.01%, NAJ was assigned to Southern 0.60%, Northern 0.33%, Asian 0.03%, and African 0.03%, and ANAJ was assigned to Southern 0.34%, Northern 0.62%, Asian 0.02%, and African 0.02% (Fig. 1b). In this case, the best K for the data based on the StructureSum algorithm [39] was 4. Two-sided two sample t-tests via Monte-Carlo permutation with 10,000 repetitions showed significant differences between AJ and NAJ for individual Northern and Southern assignment values (p < 2.2e-16 in both cases) but not for Asian (p = 0.6387) or African (p = 0.1182) assignment values (table 1).

**Table 1:** Two-sided two sample t-tests via Monte-Carlo permutation with 10000 reps showed significant differences between AJ and NAJ for Northern and Southern assignment values but not for Asian or African assignment values.

permutation of individual AJ and NAJ assignment values - 10000 reps				
	African	Asian	Northern	Southern
mean difference	0.0111	0.0027	0.4148	0.4069

p-value	0.1182	0.6387	<2.2e-16	< 2.2e-16

### Population Differentiation: grandparental country of birth

**Figure 2:**
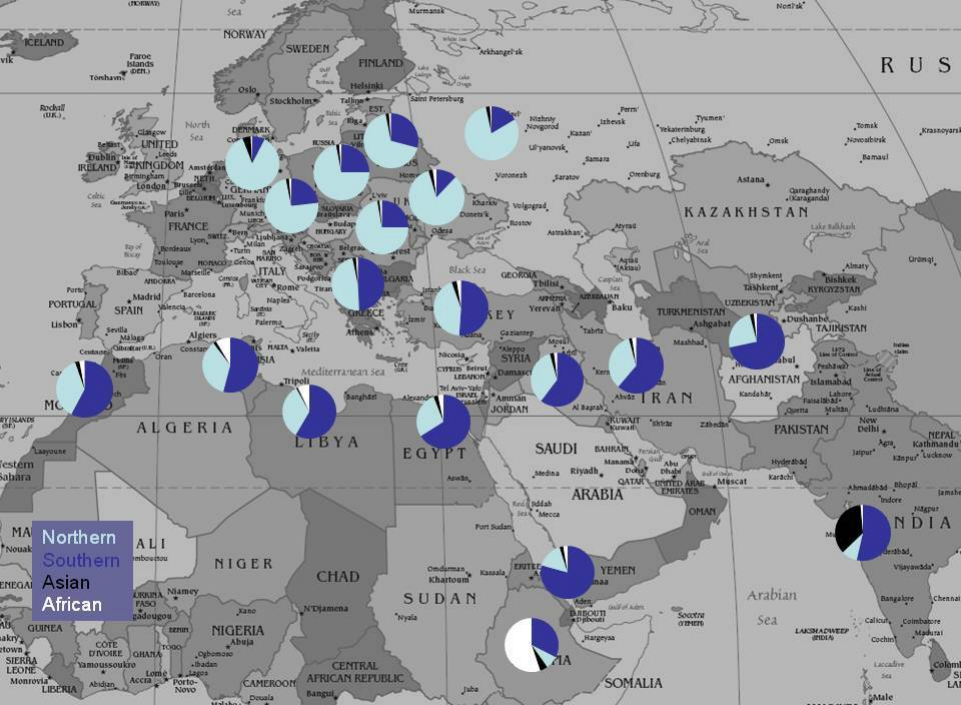
**Ancestry proportions by grandparental country of origin**. For individuals from the Jewish-Israeli sample with four grandparents from the same country and for the Ethiopian-Jewish sample, proportions of ancestry for individuals from a given geographic point are shown as segments of each pie chart.

Within the Jewish-Israeli sample, for sets of individuals reporting four grandparents from the same country of birth, we averaged percent ancestry from Northern, Southern, Asian, and African components to evaluate possible geographic influences on ancestry components. We found that, in many cases, evidence of admixture with host populations based on our autosomal data confirmed the results of previous mtDNA or Y-chromosome studies [6,20,22,23,40] (Fig. 2 and table 2). However, the variance for Northern and Southern components within each country of birth was high with the exception of individuals with all four grandparents from Germany or all four grandparents from the Ukraine.

**Table 2:** average, variance, and standard deviation of percent assignment value per cluster when K = 4

average, variance, and standard deviation of percent assignment value per cluster when K = 4													
**N**		**Southern cluster**			**Northern cluster**			**Asian cluster**			**African cluster**		

	**grandparental world region of origin (GRO)**	**ave**	**var**	**sd**	**ave**	**var**	**sd**	**ave**	**var**	**sd**	**ave**	**var**	**sd**

49	Asia	0.652	0.106	0.326	0.273	0.086	0.294	0.057	0.026	0.160	0.018	0.000	0.017

119	Africa (except South Africa, including North Africa)	0.621	0.077	0.278	0.311	0.077	0.277	0.022	0.001	0.023	0.047	0.011	0.105

44	Middle East (including Turkey)	0.606	0.087	0.296	0.341	0.087	0.296	0.025	0.001	0.027	0.028	0.003	0.054

8	Balkans (Greece, former Yugoslavia, Bulgaria)	0.493	0.133	0.365	0.474	0.138	0.372	0.021	0.000	0.017	0.012	0.000	0.013

34	Former Soviet Union	0.326	0.118	0.344	0.623	0.126	0.355	0.033	0.002	0.046	0.018	0.001	0.032

9	North America, Australia, or South Africa	0.283	0.088	0.297	0.671	0.088	0.296	0.024	0.001	0.032	0.022	0.000	0.008

115	Western, Central, or Eastern Europe	0.244	0.070	0.264	0.723	0.070	0.265	0.019	0.000	0.022	0.015	0.000	0.017

grandparental group affiliation													

230	Non-Ashkenazi	***0.602***	0.090	0.300	***0.334***	0.088	0.296	***0.031***	0.006	0.079	***0.033***	0.004	0.061

136	Ashkenazi	***0.233***	0.067	0.260	***0.729***	0.069	0.263	***0.023***	0.001	0.032	***0.014***	0.000	0.016

grandparental country of birth (GCB)													

46	Yemen	0.793	0.051	0.226	0.157	0.049	0.220	0.023	0.001	0.031	0.026	0.003	0.052

5	Afghanistan/Uzbekistan	0.716	0.079	0.281	0.243	0.075	0.274	0.023	0.001	0.023	0.017	0.000	0.008

4	Egypt	0.661	0.049	0.221	0.280	0.053	0.231	0.026	0.001	0.023	0.033	0.000	0.017

4	Bulgaria (included on Fig. 2 with Balkan)	0.629	0.105	0.324	0.329	0.109	0.330	0.029	0.000	0.019	0.013	0.000	0.008

13	Iran	0.613	0.119	0.345	0.345	0.107	0.327	0.018	0.001	0.023	0.024	0.001	0.031

29	Iraq	0.603	0.087	0.295	0.353	0.085	0.292	0.025	0.001	0.025	0.019	0.000	0.022

14	Libya	0.585	0.055	0.234	0.334	0.071	0.266	0.010	0.000	0.005	0.071	0.012	0.109

47	Morocco	0.578	0.089	0.298	0.368	0.089	0.298	0.024	0.001	0.022	0.030	0.002	0.039

10	Tunisia	0.541	0.039	0.198	0.355	0.043	0.208	0.018	0.000	0.008	0.086	0.007	0.083

6	India	0.537	0.158	0.398	0.091	0.015	0.121	0.357	0.119	0.346	0.014	0.000	0.008

5	Turkey	0.511	0.086	0.293	0.434	0.105	0.324	0.030	0.001	0.024	0.025	0.000	0.017

42	Ethiopian Jews	0.325	0.074	0.272	0.078	0.008	0.089	0.046	0.004	0.064	0.552	0.097	0.312

7	Belorus/Lithuania/Latvia/Ukraine	0.291	0.145	0.381	0.678	0.145	0.381	0.019	0.000	0.014	0.012	0.000	0.009

31	Poland	0.250	0.090	0.299	0.719	0.090	0.300	0.018	0.000	0.020	0.013	0.000	0.010

18	Romania	0.249	0.058	0.240	0.724	0.058	0.240	0.014	0.000	0.012	0.014	0.000	0.014

6	Czech/Hungary/Austria	0.231	0.056	0.237	0.737	0.056	0.237	0.018	0.000	0.016	0.014	0.000	0.005

7	Russia	0.165	0.077	0.277	0.801	0.085	0.292	0.025	0.000	0.018	0.010	0.000	0.008

6	Ukraine	0.120	*0.003*	0.055	0.834	*0.005*	0.073	0.024	0.001	0.032	0.021	0.000	0.015

6	Germany	0.075	*0.005*	0.068	0.865	*0.005*	0.067	0.050	0.002	0.048	0.010	0.000	0.002

Shown, for a given subset of individuals, are average, variance, and s.d. of assignment values from STRUCTURE results when K = 4. Differences in assignment values for Northern, Southern, Asian, and African clusters (italicized and in bold) were found to be significant between Non-Ashkenazi and Ashkenazi Jews based on 10000 permutations of assignment values. Variance of Northern and Southern assignment values was high within a subset based on grandparental country of birth (GCB), with the exception of Ukraine and Germany (italicized). For GCB, in four instances, individuals were combined into a subset when GCBs were adjoining geographically and had a history of border shifts and periods of unification or division (ex: Czech/Hungary/Austria), such that the town or city in which a grandparent was born might currently lie in one of the neighboring countries rather than the stated GCB.

### Hardy Weinberg Equilibrium (HWE)

No population showed significant deviation from HWE expectations over all loci. Following application of a Bonferroni correction to correct for multiple testing (requiring a p value of 0.05/32 = 0.00156 for significance), no p-values for individual loci are significant.

### Marker Information Content

The tetranucleotide and dinucleotide markers had similar average non-Ashkenazi/Ashkenazi delta values (0.133 and 0.130, respectively). Overall, the delta values for this marker panel (0.131) would not indicate a robust ability to differentiate between these two populations but the results of this study (considering the consistency of the observed ancestry coefficients with known geography and previous studies) show their utility for this purpose nonetheless (table 3).

**Table 3:** Delta values for each population pair for each marker

	Delta values for each population pair													
**Locus**	**EJ/ANAJ**	**EJ/NAJ**	**EJ/AA**	**EJ/EA**	**AJ/ANAJ**	**AJ/NAJ**	**AJ/AA**	**AJ/EA**	**ANAJ/NAJ**	**ANAJ/AA**	**ANAJ/EA**	**NAJ/AA**	**NAJ/EA**	**AA/EA**

**CSF1PO**	0.09	0.10	0.18	0.18	0.13	0.05	0.16	0.11	0.09	0.22	0.19	0.17	0.10	0.15

**D2S1338**	0.30	0.25	0.32	0.37	0.10	0.18	0.26	0.11	0.14	0.24	0.12	0.29	0.14	0.28

**D3S1358**	0.14	0.10	0.18	0.18	0.17	0.17	0.16	0.11	0.12	0.12	0.18	0.20	0.14	0.21

**D5S818**	0.22	0.11	0.21	0.13	0.15	0.12	0.18	0.12	0.21	0.20	0.24	0.17	0.14	0.18

**D7S820**	0.16	0.09	0.16	0.09	0.11	0.05	0.18	0.08	0.08	0.26	0.14	0.19	0.07	0.13

**D8S1179**	0.19	0.14	0.30	0.28	0.10	0.16	0.27	0.16	0.13	0.25	0.10	0.30	0.18	0.30

**D13S317**	0.23	0.12	0.18	0.19	0.05	0.17	0.33	0.11	0.13	0.33	0.10	0.22	0.08	0.22

**D16S539**	0.18	0.11	0.22	0.12	0.07	0.11	0.19	0.07	0.17	0.22	0.10	0.15	0.11	0.23

**D18S51**	0.28	0.28	0.29	0.22	0.12	0.18	0.40	0.19	0.15	0.40	0.19	0.43	0.20	0.24

**D19S433**	0.21	0.15	0.25	0.22	0.12	0.14	0.24	0.07	0.15	0.25	0.10	0.17	0.13	0.25

**D21S11**	0.30	0.26	0.34	0.41	0.22	0.13	0.28	0.23	0.16	0.26	0.25	0.29	0.21	0.21

**FGA**	0.24	0.19	0.20	0.20	0.13	0.13	0.27	0.10	0.19	0.35	0.17	0.20	0.15	0.27

**TH01**	0.26	0.19	0.19	0.20	0.05	0.19	0.36	0.13	0.17	0.33	0.10	0.29	0.13	0.24

**TPOX**	0.24	0.23	0.22	0.24	0.07	0.05	0.20	0.08	0.05	0.25	0.08	0.23	0.06	0.28

**vWA**	0.24	0.16	0.20	0.20	0.10	0.17	0.35	0.12	0.14	0.38	0.11	0.27	0.10	0.36

**D17S799**	0.35	0.42	0.24	0.45	0.14	0.12	0.52	0.19	0.10	0.41	0.21	0.49	0.12	0.51

**D8S272**	N/A	N/A	N/A	N/A	0.09	0.18	0.34	0.10	0.17	0.35	0.17	0.46	0.18	0.34

**D7S640**	0.35	0.36	0.42	0.41	0.15	0.15	0.40	0.17	0.16	0.43	0.20	0.32	0.19	0.35

**D8S1827**	0.21	0.21	0.36	0.20	0.09	0.09	0.42	0.11	0.03	0.50	0.18	0.50	0.17	0.38

**D22S274**	0.16	0.15	0.19	0.24	0.15	0.09	0.16	0.12	0.14	0.13	0.18	0.14	0.16	0.20

**D5S407**	0.20	0.18	0.27	0.31	0.08	0.14	0.34	0.18	0.17	0.35	0.20	0.26	0.18	0.32

**D2S162**	0.27	0.26	0.34	0.33	0.16	0.17	0.37	0.12	0.19	0.40	0.22	0.30	0.21	0.46

**D10S197**	0.34	0.32	0.31	0.26	0.13	0.22	0.26	0.15	0.11	0.30	0.11	0.31	0.09	0.23

**D11S935**	0.23	0.18	0.44	0.25	0.07	0.12	0.65	0.18	0.07	0.64	0.16	0.59	0.14	0.58

**D9S175**	0.33	0.30	0.50	0.34	0.17	0.13	0.46	0.13	0.17	0.54	0.19	0.49	0.12	0.49

**D5S410**	0.21	0.13	0.24	0.27	0.10	0.10	0.43	0.16	0.12	0.42	0.15	0.34	0.22	0.44

**D7S2469**	0.29	0.23	0.30	0.29	0.15	0.10	0.31	0.13	0.12	0.31	0.15	0.32	0.14	0.33

**D16S3017**	0.28	0.23	0.21	0.30	0.18	0.13	0.27	0.08	0.09	0.32	0.10	0.28	0.09	0.30

**D10S1786**	0.31	0.24	0.27	0.28	0.09	0.12	0.49	0.05	0.10	0.54	0.09	0.49	0.13	0.48

**D15S1002**	0.23	0.12	0.38	0.21	0.13	0.13	0.53	0.12	0.14	0.51	0.14	0.43	0.12	0.48

**D6S1610**	0.26	0.19	0.15	0.18	0.12	0.13	0.28	0.16	0.13	0.31	0.24	0.24	0.16	0.25

**D1S2628**	0.32	0.27	0.28	0.35	0.07	0.10	0.46	0.08	0.13	0.49	0.06	0.39	0.11	0.45

**AVERAGE**	0.25	0.20	0.27	0.25	0.12	0.13	0.33	0.13	0.13	0.34	0.15	0.31	0.14	0.32

## Discussion

Using 32 autosomal STR markers and the program STRUCTURE, we differentiated between Ashkenazi and non-Ashkenazi Jewish populations in the form of Northern and Southern genetic components. We also demonstrated the utility of including reference populations when attempting to detect population substructure within closely related populations. Notably, we revealed substructure among Jews using a small STR panel, but only when additional samples with ancestry from African, Asian, and European continental populations were included in analyses. The recent study by Kopelman et al [28] used genotypic data from considerably more STRs than our study; however, we found that inclusion of additional populations and high information content per marker apparently compensated, in part, for the relatively low number of markers we used. We also suggest that the clustering patterns in their study may have been somewhat altered if they had not, in effect, assumed that Jewish populations were a product of Middle Eastern and European ancestry, only. Our results indicate that only with the inclusion of world-wide samples is it possible to infer proportions of world-wide ancestry in a highly migratory sample such as Jews with grandparents born on all continents.

Xu and Jin [41] demonstrated both European and Asian contributions to the Uyghur population of Western China when STRUCTURE and PCA were used to analyze European, East Asian and Uyghur sample data. They noted, however, that when world-wide HGDP-CEPH samples including Uyghur were analyzed in other studies, there appeared to be three parental populations for Uyghur: European, East Asian, and Central Asian [42,43]. Consistent with our results, this is an example of the value of including additional reference samples or parental populations in detecting subtle substructure and admixture in the populations to which they contributed.

Our finding that there is little Sub-Saharan African admixture in North African Jewish populations (average percent African ancestry component based on STRUCTURE results for samples with four grandparents from a given country: Libya (0.07), Morocco (0.03), Tunisia (0.09), Egypt (0.03)) are consistent with findings from the Behar mtDNA study, which detected low rates of Sub-Saharan African, and no North African maternal contribution to Moroccan, Tunisian, and Libyan Jewish populations. Our findings of significant Sub-Saharan African ancestry in Ethiopian Jews (0.55) in contrast to Yemenite Jews (0.03) were also consistent with mtDNA [26] and Y-chromosomal [23] studies. Our analyses based on autosomal data cannot rule out local North African contribution to the North African Jewish populations studied here. However, given the finding that non-Jewish North African populations have approximately 25% [44] Sub-Saharan African mtDNA contribution, we would have expected a significant Sub-Saharan component in the North African Jewish populations that we included. That we did not find such a component may reflect relative reproductive isolation among the North African Jewish communities and their host populations.

Our results on population substructure reflect the influence of numerous factors, including the recent founding of Ashkenazi vs. non-Ashkenazi Jewry, gene flow between these groups and between Jewish and non-Jewish populations, a highly complex migration history, and the characteristics and limitations of the marker set used in this study. The documented history of the Eurasian and North African Jewish populations indicates that the Diaspora did not radiate outward geographically from the Middle East in a simple starburst pattern -- rather, Ashkenazi and non-Ashkenazi Jews migrated repeatedly in and out of Europe, Africa, the Middle East, and Central, South, and East Asia. Cultural differences, once established, may have promoted differentiation between the Ashkenazi and non-Ashkenazi Jews in spite of their repeated geographic overlap. High variance of Northern and Southern components within a subset of individuals with the same grandparental country of birth even for those with large sample size (table 2) likely reflects recent admixture [41] as well as the small set of markers.

Although within each group there is a high degree of variability among individual assignment values, geographic patterns are seen in the average North/South percent assignment values between groups as defined by AJ or NAJ, grandparental world region of birth, or grandparental country of birth. For AJ and NAJ these differences were found to be statistically significant (table 1) (significance was not tested for differences between regions or grandparental country of birth because sample sizes varied greatly). Thus, even based on data from a small marker set, AJ are not a homogeneous population. For non-Ashkenazi Jews, the small measured Sub-Saharan and small inferred Northern African contribution in all Jewish communities of African origin other than Ethiopian may be due to a greater degree of endogamy within those communities.

After demonstrating the feasibility of distinguishing Ashkenazi Jews from non-Jewish Europeans using autosomal SNPs, Need et al [10] analyzed these samples in conjunction with a number of Middle-Eastern populations and concluded, in contrast to Behar et al [6], that the differentiation of Ashkenazi Jews from non-Jewish Europeans was due to their Middle-Eastern ancestry rather than a bottleneck event because the Ashkenazi Jewish sample had high heterozygosity. Although we agree that the Middle-Eastern ancestry of Ashkenazi Jews is demonstrated by the PCA analyses in Need et al [10], we are less confident in the validity of their conclusion that no bottleneck occurred. As we demonstrate, there is wide genetic variation among Ashkenazi Jewish sub-populations both between and within grandparental country of birth. Elevated heterozygosity may reflect either recent admixture or the combining of multiple Ashkenazi populations by Need et al into one sample for analysis.

Our autosomal data from a small number of Jews with grandparents from India (N = 6) show significant Asian admixture (0.36) while also showing the highest ratio of Southern/Northern contribution. Of all non-Ashkenazi Jewish populations, Jews of Yemenite, Egyptian, and Central Asian descent have the largest Southern component (0.79, 0.73, and 0.72, respectively). These results concur with both molecular and historical evidence. Behar et al [6] studied a large mtDNA dataset of non-Ashkenazi Jews and their previous dataset of Ashkenazi Jews and detected a small amount of Sub-Saharan African and no local North African maternal contribution to Moroccan, Tunisian, and Libyan Jewish populations. Sub-Saharan African-specific mtDNA lineages were found at high frequencies in Ethiopian and moderate frequencies in Yemenite Jews. Local South Asian mtDNA contribution was detected in two Indian Jewish populations. Historically, Yemenite, Egyptian, and Central Asian are some of the oldest Jewish communities, established after the first Jewish exile from Jerusalem [45-48]. Jews settled in India as early as the 7^th ^century CE, possibly from Iran or Yemen and incorporated local residents as well as slaves into the population [48].

### Theoretical implications

The large Jewish-Israeli sample in this study was collected as part of a greater study on health-related phenotypes; it contained 526 individuals with grandparents from a broad geographic range: Northern and Southern Europe, Russia, North Africa, Ethiopia, the Middle East, Central Asia, and India. While it is not surprising that this sample (based on historical accounts, mtDNA and Y-chromosome studies, and geographic range) has components of all three major continental populations that vary based on recent ancestral origin, these differences in ancestry were not detected without the addition of putative parental population or reference samples in STRUCTURE analysis. The significance of the resultant ancestry components could not have been evaluated in the absence of self-reported information on family history and identification. Such detail is not always available for population samples; however it proved to be highly valuable in this case. Others have shown that the ability to detect population substructure is dependant, in part, on sample size or the inclusion of reference populations [41,49,50]. For a sample in which populations have diverged recently or have low levels of genetic differentiation (such as Ashkenazi and non-Ashkenazi Jews), the ability to detect substructure increases with the amount of data available, with the total data being a result of information derived both from the number and informativeness of *samples *as well as the number and informativeness of markers [49,51].

This issue has great practical relevance for the substructure testing phase of association mapping studies in which cases and controls are from the same self-identified population group, particularly when increasing the number of AIMs is not an immediate option. Numerous other genetic studies have shown that Jewish populations, while sharing ancient Middle-Eastern ancestry, have practiced exogamy or incorporated members of local populations to some extent [3-6, 8, 11, 20, 22, 23]. It is this gene flow from host populations combined with genetic drift and possible local selection pressures, that have led to detectable substructure among Jewish populations, perhaps more so than would be expected based solely on time since population divergence.

### Historical Implications

In contrast to Seldin et al [25], we showed varying Northern and Southern components among Ashkenazi Jewish populations. Non-Jewish population samples in the Seldin et al study were European or European American in origin, while our study included African-American and Asian samples as well as European Americans. In addition, the Jewish sample in Seldin et al included only three Sephardic (based on their nomenclature) Jews, too few to provide reliable information about this population. The Kopelman et al study [28], in addition to European Jews and non-Jews, included two North African Jewish populations as well as Middle Eastern non-Jewish populations. Our study included a large number of non-Ashkenazi Jews including those from North Africa, the Near East, Ethiopia, and Central and South Asia. We believe that our study demonstrates the effect of *relative *population genetic differences and total information from markers and individuals on clustering patterns. As we have described previously, our STR panel was chosen specifically for its high information content [50]. Although the major consideration in marker selection for this panel was the ability to differentiate between major American populations, we previously demonstrated that the same panel was, somewhat unexpectedly, also very useful at distinguishing different Asian populations [52].

The migratory history and origins of Ashkenazi Jews are less clear than those of non-Ashkenazi Jews. During the early Middle Ages in Europe, Jews lived in close proximity with their non-Jewish neighbors in small villages with constant interaction. Intermarriage, although periodically outlawed by host-country governments, occurred with some regularity [53]. In addition, Jewish Europe was never solely inhabited by Ashkenazi Jews. Some Jews expelled from Spain during the inquisition settled in part of the Ottoman Empire, which includes the Balkans (present-day Turkey, Bulgaria, Greece, Bosnia, and Serbia), while others went to Italy, Holland, and France [54]. In fact, all subjects in this study who identified their grandparents as having come from Balkan countries also identified themselves as non-Ashkenazi and those with two grandparents from Balkan countries identified the parent on that side as non-Ashkenazi.

We believe that the apparent Southern genetic component of those of European descent (Jewish or not), as well as that seen in Jews, is actually originally Middle Eastern in origin. This is consistent to various degrees with previous results from y-chromosomal [3,8], mtDNA [3,5,21] and autosomal evidence [25,26] as well as historical evidence. The large Northern component in all Ashkenazi populations included in this study indicates significant local contribution to these populations, which either occurred early in their histories (German and Ukraine) or in small increments over time (other Ashkenazi populations as evidenced by high variance of Northern/Southern components). Among non-Ashkenazi Jewish populations sampled, although all have exhibited porous membership, Ethiopian and Indian Jewish communities have particularly significant local contributions to their gene pools.

The high variance for percent Southern and Northern components for group affiliation, region of birth, and grandparental country of birth, indicate that admixture and migratory events are recent [41]. This reflects both the complex migration histories of Jewish populations and the limitations of the marker set used here, including the possibility of homoplasious alleles interfering with accurate ancestral population assignment. Despite small sample sizes, the variance was small for individuals with four German (N = 6) (Southern σ^2 ^= 0.005, Northern σ^2 ^= 0.005) or four Ukrainian (N = 6) (Southern σ^2 ^= 0.003, Northern σ^2 ^= 0.005) grandparents, which may indicate that admixture events in these populations are older than those of other Jewish populations in our study.

## Conclusions

Our results reinforce conclusions of previous characterizations of Jewish samples based on uniparentally-inherited segments of the genome. Jewish populations are not necessarily genetically homogeneous, either as a whole, within the Ashkenazi or non-Ashkenazi affiliations, or within a continent. Geographic gradients of genetic heterogeneity such as that observed here within what is seemingly one population have been shown empirically to confound association studies [26], but in the absence of a very large AIM panel, are correctable when information such as grandparental country or region of birth is used to create subsets of matched cases and controls [55,56].

Although clustering programs such as STRUCTURE are designed to assign proportions of ancestry to individuals without the necessity of including parental population information, when our mixed Jewish sample was analyzed without the EA, AA, Thai, and Chinese samples, the substructure within Jews was not apparent. While it is true that Jewish samples would be shown to contain substructure if analyzed with thousands of SNP markers or hundreds of STR markers, it is unlikely that subtleties contributed by Asian and African admixture would be detected without inclusion of world-wide reference samples. For example, Kopelman et al [28] used data from 678 STRs for four Jewish populations (Moroccan, Tunisian, Turkish, and Ashkenazi) combined with that of Middle Eastern and European populations and it was found that the Jewish populations had ancestry to varying degrees from both European and Middle Eastern populations. They do not find information regarding Asian or Sub-Saharan African admixture because that is not possible without the inclusion of samples from those regions. When they used STRUCTURE to analyze their Jewish samples, alone, the best fit for the data was two parental populations.

We demonstrated empirically, the effect of reference population inclusion on the ability to cluster individuals in an admixed population. Studies commonly control for population stratification by genotyping subjects, only, with a panel of non-coding markers. However, when cases and controls have been matched (non-genetically) for ancestry and no other populations that could potentially contribute to admixture are included in the analysis, any existing substructure is unlikely to be detected. We suggest that samples of reference or proxy parental populations be included in the substructure testing phase of case control association studies when the participants are sampled from potentially admixed populations such as populations residing in or originating from major human migratory pathways, urban populations, or American populations.

The total number of markers used in this study is quite small in comparison to many other available studies, but due to higher mutation rates and number of alleles per locus STRs provide much more information, on average, than SNPs for population assignment and population stratification [19]. The high variation and high mutation rates for STRs may backfire, however, when attempting to distinguish between populations that have diverged long ago, as homoplasic alleles can accumulate under those circumstances. We found previously [52] that the tetranucleotide CODIS loci were not useful in distinguishing between AA and EA populations while they were highly informative when distinguishing among more recently-diverged Asian minority populations [57]. This marker set may be more useful for detecting recent admixture or founding events, such as those which formed the Jewish populations in question, here.

## Methods

### Populations and Sampling

A total of 780 subjects were selected for inclusion in this study: mixed Jewish (N= 526) (the central sample of interest for which it was our goal to detect population structure), Ethiopian Jewish (EJ, N= 42), Thai (N = 45), Chinese (N = 29), African American (AA, N = 54), and European American (EA, N = 91). For some analyses, the mixed Jewish sample, collected in Israel, was divided into subsets based on self-reported information about parents. This included individuals with two Ashkenazi parents (AJ, N = 135), individuals with two non-Ashkenazi parents (NAJ, N = 226), and individuals with one Ashkenazi and one non-Ashkenazi parent (ANAJ, N = 38). These sub-samples do not add up to the total mixed Jewish sample due to missing self-report parental or grandparental information. The Asian populations in this study were collected as part of an ongoing gene mapping study. Samples of individuals self-identified as being of Thai and Chinese ancestry were obtained from a blood drive in Bangkok, Thailand. The Thai and Chinese samples used in this study were selected to include only subjects for whom all four grandparents were reported to have the same self-identified ethnicity as the subject. For analyses the Thai and Chinese samples were combined into one sample labeled Asian. The dataset also included samples of unrelated AAs and EAs a subset of a sample described elsewhere and for which population group self-identifications were previously confirmed via Bayesian marker clustering [50]. Note that the EA sample was not screened to exclude AJ or NAJ subjects so it is likely to contain small numbers of them. The EJ sample was obtained from the National Laboratory for the Genetics of Israeli Populations, Sackler Faculty of Medicine, Tel Aviv University, Israel. This work was approved by the Yale University School of Medicine Human Investigation Committee HIC#12183, New York State Psychiatric Institute Institutional Review Board protocol#4753, Israel Board of the Ministry of Health Helsinki Committee for Genetic Trials #920050036, and the Department of Veterans Affairs Subcommittee on Human Studies #0008. All subjects provided informed consent as approved by the appropriate institutional review boards.

### Markers and Genotyping

All samples were genotyped for thirty-two unlinked autosomal STR markers (with the exception of EJ, for which data are missing for D8S272). The panel is comprised of the 15 tetranucleotide repeats in the AmpF/STR Identifiler PCR Amplification kit (PE Applied Biosystems, Foster City, CA, USA) (D8S1179 [GenBank:AX412206], D21S11 [GenBank:AJ550387], D7S820 [GenBank:NC_000007], CSF1PO [GenBank:AF076965], D3S1358 [UniSTS:148226], TH01 [UniSTS:240639], D13S317 [GenBank:G09017], D16S539 [GenBank:AF249681, D2S1338 [GenBank:G08202], D19S433 [GenBank:G08036 ], vWA [UniSTS:240641], TPOX [GenBank:M25706], D18S51 [GenBank:L18333 ], D5S818 [GenBank:G08446 and FGA [GenBank:G3347]) and an additional 17 dinucleotide repeats (D17S799 [GenBank:Z16830], D8S272 [GenBank:Z17250], D7S640 [GenBank:Z23671], D8S1827 [GenBank:Z50970], D22S274 [GenBank:Z16730, D5S407 [GenBank:Z16723], D2S162 [GenBank:Z17035], D10S197 [GenBank:Z16611], D11S935 [GenBank:Z17148], D9S175 [GenBank:Z17021], D5S410 [GenBank:Z16825], D7S2469 [GenBank:Z53000], D16S3017 [GenBank:Z52036], D10S1786 [GenBank:Z51854], D15S1002 [GenBank:Z53249], D6S1610 [GenBank:Z53131], and D1S2628 [GenBank:Z52173]). The amelogenin locus, included in the AmpF/STR Identifiler PCR Amplification kit for sex identification, was not included in any analyses. All STR markers were analyzed on an ABI PRISM 3100 semiautomated capillary fluorescence sequencer. Data were scored using Genemapper (ABI). We previously used this marker panel (with the addition of D1S196, D2S319, D7S657, D12S352, D14S68, which were not used here either because they were replaced with D7S2469 and D1S2628 or because of a large number of failed genotypes) to determine and statistically correct for ancestry in case-control studies and genome-wide linkage studies [58-61] and in population genetics studies [52].

### Statistical Analyses

#### Population Differentiation

The program STRUCTURE 2.2 [36,37] uses Bayesian clustering of multilocus genotypes to assign individuals to populations, estimate admixture proportions for individuals, and infer the number of parental populations (K) for a sample. Because variance of STRUCTURE results increases with small sample sizes each run was repeated 5 times with all 32 STR markers in the panel. For analyses that included AA, EA, Thai, Chinese, EJ and mixed Jewish populations, the parameters used were K = 2 through K = 9 and 50,000 burn-in and 50,000 Markov chain Monte Carlo (MCMC) iterations. For analyses that included only the EA and mixed Jewish samples in the absence of all other samples, the parameters used were K = 2 through K = 4 and 50,000 burn-in and 50,000 Markov chain Monte Carlo (MCMC) iterations. The self-reported population of origin was not used as additional data by STRUCTURE and the presence of admixture was assumed.

The authors of STRUCTURE recommend using the maximal value for lnP(D) to determine the best value of K for the data. However, it has been observed that lnP(D) will plateau while continuing to increase slightly as assumed K increases past the correct K. Therefore, identifying the K for which lnP(D) is greatest may not be sufficient to identify the correct (underlying) K. We employed StructureSum, an R script that uses the output from STRUCTURE to identify the K for which lnP(D) is maximized while both |lnP(D)_K+1 _- (lnP(D)_K _- lnP(D)_K-1_)| and variance of lnP(D)are minimized. This identifies the highest value of K, prior to the plateau of lnP(D) [38].

STRUCTURE runs were unsupervised, using the admixture model and correlated allele frequencies. Structure randomly assigns clusters in each run such that the correspondence between runs is non-obvious. CLUMPP software [62] takes the multiple results files and determines which clusters from different runs correspond, then averages the assignment values between runs for each individual. To account for cluster label switching between runs, we used the fullsearch option and non-weighted alignment procedure in CLUMPP version 1.1.1 to identify corresponding clusters between runs for a set of five runs with a given K and to produce average membership coefficients for each individual for each cluster. These average assignment values were used with the program, DISTRUCT [63], to produce graphs of STRUCTURE output.

For Jewish-Israelis with four grandparents from the same country, for K = 4, individual assignment values produced by CLUMPP were averaged to arrive at values for Northern, Southern, Asian, and African ancestral components for the Jewish population of that geographic location. This was also done for AJ, NAJ, and ANAJ.

A two Sample t-test via monte-carlo permutation was used to test for significance between the individual AJ and NAJ assignment values with 10,000 samples simulated when H_0 _(no significant difference between average assignment values for two populations) is true. The t-test value of the observed data was then compared to that of the simulated data to obtain a p-value for mean differences in assignment values between AJ and NAJ.

#### Hardy Weinberg Equilibrium (HWE)

Tests for deviation from Hardy-Weinberg equilibrium expectations were conducted using GENEPOP 4.0 [64] globally for all loci using sub-option 5, the exact test for HWE in which H_1 _= heterozygote excess based on a Markov chain method. The parameters used were 5000 dememorizations, 1000 batches, and 5000 iterations per batch. The parameter values were increased from defaults until the observed standard error for p-values was less than 0.01. For the mixed Jewish sample this was performed for the sample as a whole, as well as for the AJ, NAJ, and ANAJ subsets. We used an exact test for multi-allelic markers because Chi-squared tests are inappropriate for such analyses [65].

#### Marker Information Content

Markers were evaluated for delta (δ) [66], a measure of marker information content, reflecting the ability of a marker to differentiate statistically between populations. We have confirmed that this is a relevant measure for the markers we employed herein [50]. To arrive at δ, the absolute values of allelewise frequency differences between two populations are added and this sum is divided in half, i.e.,  where  and  are the allele frequencies for the *i*^th ^allele in populations A and B. The more effective the marker is at differentiating between populations, the higher the value for δ [50].

## Authors' contributions

JBL designed the study, carried out statistical analyses and drafted the manuscript. DH participated in project coordination, sample collection, and the writing of the manuscript. AS and AM participated in sample collection in Thailand. HRK carried out sample collection in the United States. RTM participated in project coordination and sample collection. EA and BS participated in project coordination and sample collection in Israel. JG participated in study design and supervision, project coordination, sample collection, and the writing of the manuscript. All authors read and approved the final manuscript.
